# Supplementary material for: E(3)-equivariant graph neural networks for data-efficient and accurate interatomic potentials
Source: Nat Commun. 2022 May 4;13:2453. doi: 10.1038/s41467-022-29939-5 (PMC9068614; doi:10.1038/s41467-022-29939-5)
Supplement: Supplementary file 1 — Supplementary Information [file 41467_2022_29939_MOESM1_ESM.pdf]

Supplementary Information

E(3)-Equivariant Graph Neural Networks for Data-Efficient and Accurate Interatomic Potentials

S. Batzner et al.

Supplementary Figures

Long Molecular Dynamics Simulation of $Li_4P_2O_7$

Figure 1 shows the Radial Distribution Function obtained from a MD simulation of simulation length 500ps compared to the AIMD simulation of 50ps. For both simulations, there first 10ps were not used in the computation of the RDF.

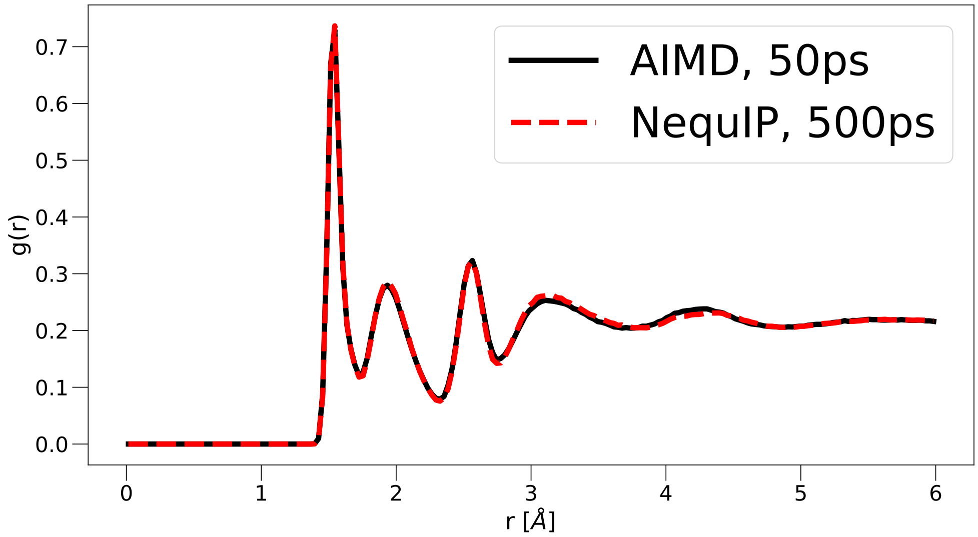

Supplementary Figure 1: RDF of $Li_4P_2O_7$ of 500ps NequIP simulation with 50ps AIMD simulation.

Learning Curves

Figure 2 show the force MAE of different NequIP models. as a function of data set size for the aspirin molecule in MD-17. Figure 3 shows force errors as a function of training set size on the aspirin molecule in the MD-17 data set, together with weight- and feature-controlled version of the $l = 0$ network. Figure 4 shows energy errors as a function of training set size on the data set from [1].

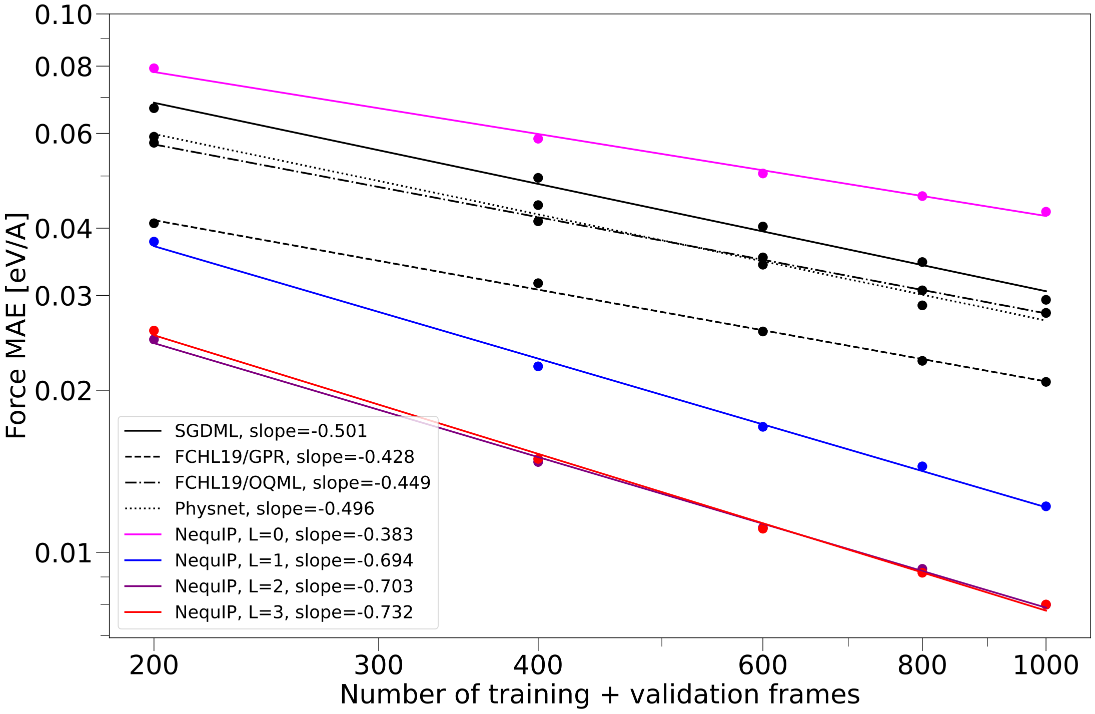

Supplementary Figure 2: Log-log plot of the predictive error on the aspirin molecule in MD-17 using NequIP with $l \in \{0, 1, 2, 3\}$ as a function of data set size used for training and validation, measured via the force MAE.

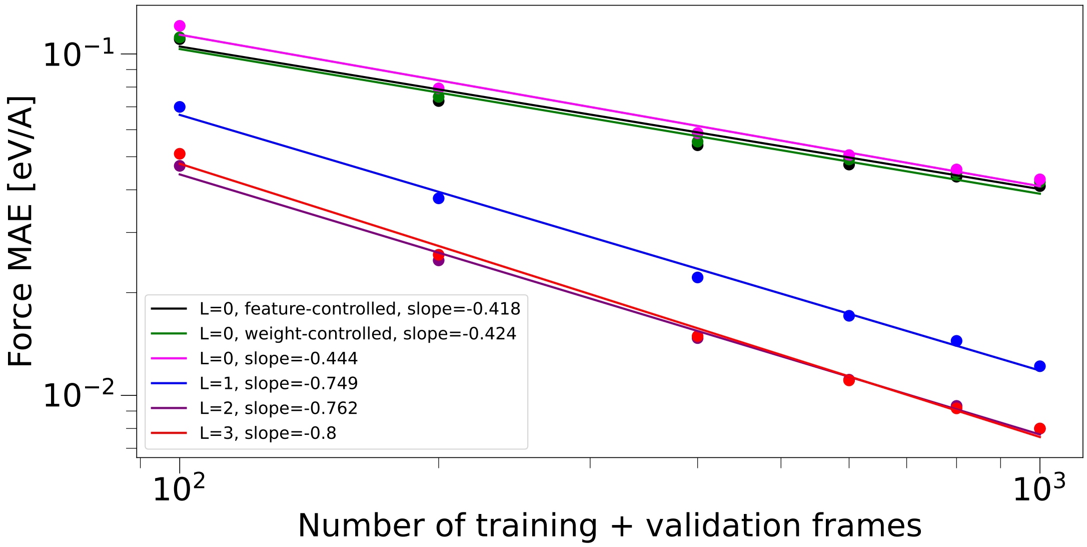

Supplementary Figure 3: Log-log plot of the predictive error on the aspirin molecule in MD-17 using NequIP with $l \in \{0, 1, 2, 3\}$ as a function of data set size, measured via the force MAE. The plots also shows the weight- and feature-controlled version of NequIP.

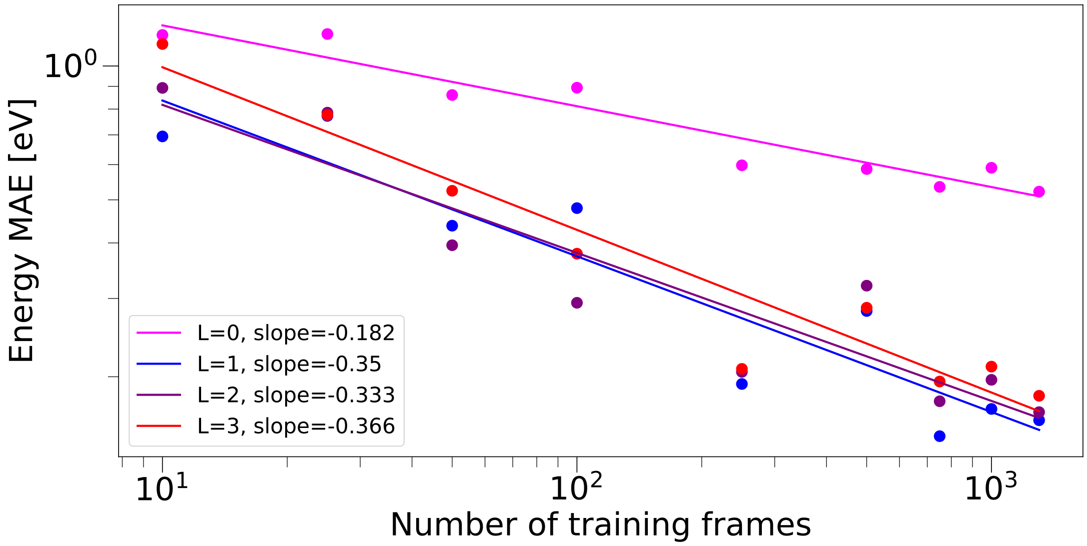

Supplementary Figure 4: Log-log plot of the predictive error on the water data set from [1] using NequIP with $l \in \{0, 1, 2, 3\}$ as a function of training set size, measured via the energy MAE.

Revised MD-17 data set

Figures 5 and 6 show histograms of the energy and force labels on aspirin in the revised MD-17 data set, respectively.

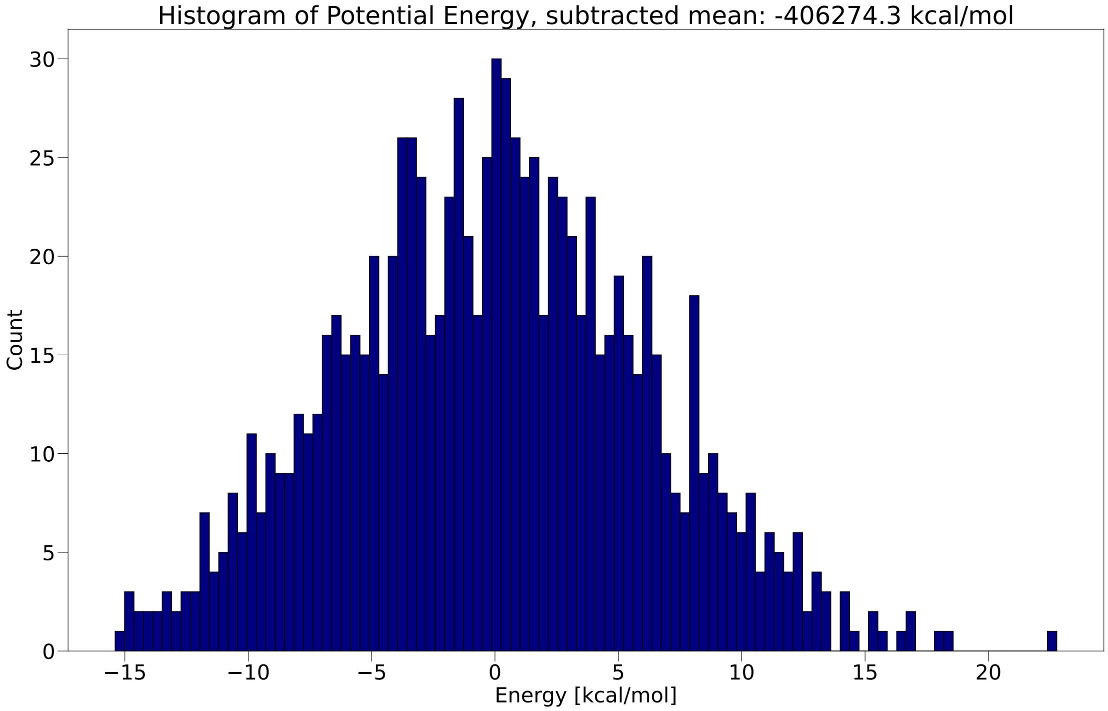

Supplementary Figure 5: Histogram of potential energies of all structures used for training and validation for the aspirin molecule in the revised MD-17 data set. The mean energy was subtracted before plotting.

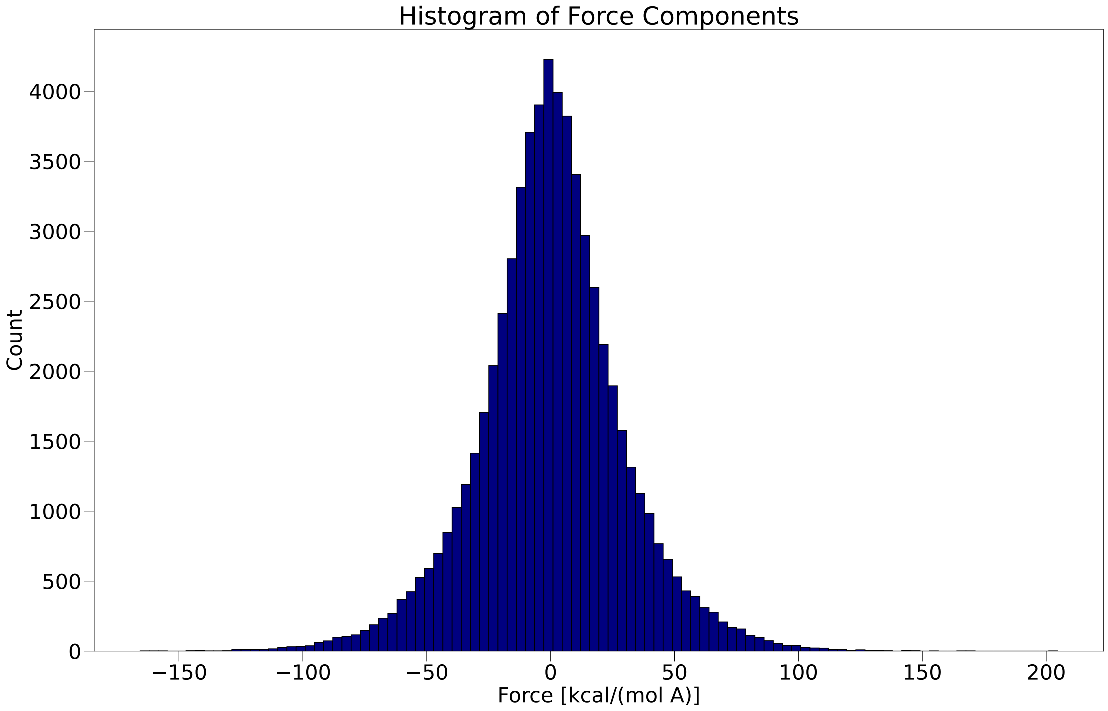

Supplementary Figure 6: Histogram of force components of all structures used for training and validation for the aspirin molecule in the revised MD-17 data set.

Supplementary Tables

Results on CCSD/CCSD(T) Reference Data

Molecule		sGDML	GemNet-(T/Q)	NequIP (l=3)
Aspirin	*Energy*	6.9	-	**2.0**
	Forces	33.0	10.3	**8.1**
Benzene	*Energy*	0.17	-	**0.05**
	Forces	1.7	0.7	**0.26**
Ethanol	*Energy*	2.2	-	**0.36**
	Forces	15.2	3.1	**2.5**
Malonaldehyde	*Energy*	2.6	-	**0.72**
	Forces	16.0	5.9	**4.4**
Toluene	*Energy*	1.3	-	**0.27**
	Forces	9.1	2.7	**1.7**

Supplementary Table 1: Energy and Force MAE for molecules at CCSD/CCSD(T) accuracy, reported in units of [meV] and [meV/Å], respectively, and a training budget of 1,000 reference configurations. For GemNet, the best result out of the T/Q versions is presented.

Supplementary References

[1] Cheng, B., Engel, E. A., Behler, J., Dellago, C. & Ceriotti, M. Ab initio thermodynamics of liquid and solid water. *Proceedings of the National Academy of Sciences* **116**, 1110–1115 (2019).
